# Brain metastases epidemiology in a Tunisian population: trends and outcome

**DOI:** 10.2217/cns-2017-0020

**Published:** 2018-01-19

**Authors:** Mehdi Benna, Nesrine Mejri, Manel Mabrouk, Houda El Benna, Soumaya Labidi, Nouha Daoud, Hamouda Boussen

**Affiliations:** 1 Medical Oncology Department, Abderrahmen Mami Hospital, Ariana, Tunisia

**Keywords:** brain metastases, epidemiology, survival

## Abstract

**Aim:**

We reported anatomo-clinical features of brain metastases (BMs) collected in a Tunisian medical oncology department.

**Patients & methods:**

We retrospectively identified all cases of BM within a cohort of 7055 patients, treated for a histologically confirmed nonhematological cancer between 2000 and 2016. Data about age, sex and primary tumor were collected.

**Results:**

Incidence was 1.9% and mean age was 54 years with a 1.24 sex ratio. BMs were symptomatic in 73.7% of cases after a median time of 16 months. A total of 73.4% patients receiving local therapy, 88% by whole brain radiation therapy and 21.6% had a metastasectomy. Lung and breast cancers were the primary in 80% of the BM.

**Conclusion:**

BM showed trends of young with underestimated incidence.

**Summary points**

- Brain metastases (BM) are becoming an increasing burden on the health care system with improvement of cancer patients survival.
- We reported in our study epidemiological patterns and outcomes of BM from solid tumors in everyday clinical practice.
- We observed an incidence of 1.9% which is likely under estimated and showed a predominance of lung and breast cancer primaries.
- We also observed a younger age at diagnosis and better survival in breast cancer patients, which is different from what is reported in the literature.

Brain metastases (BMs) from solid tumors represent the most frequent intracranial tumors [1]. Metastases can occur in the brain parenchyma and/or spinal cord and/or leptomeninges [2,3]. With the improvement of cancer management, the increased survival in metastatic patients led to an increase in the number of patients with BM. The incidence varies according to the study type, based on clinical, surgical or autopsic series. It is also reported that incidence is underestimated and keeps increasing; ‘thanks’ to higher resources utilization and more complex treatment algorithms [4,5]. Survival of cancer after BM is poor no matter how vigorous the treatment is [6]. The most important data about BM investigates the integration of modern diagnostic techniques or the development of new therapeutic approach. Descriptive studies are important to better understand the natural history of cancer and better guide personalized therapeutic approaches. In Tunisia, as in North Africa, incidence and epidemiological characteristics of cancer is slightly different but getting closer to trends observed in Europe and the USA [7]. However, data about BM from solid tumors in this population are missing. Such data are needed since diagnosis and treatment of BM have become an increasing burden on the healthcare system spanning across many medical subspecialties.

We reported in our study epidemiological patterns and outcome of BM from solid tumors in everyday clinical practice.

## Patients & methods

We retrospectively analyzed a cohort of 7055 patients, diagnosed with histologically confirmed cancer treated in medical oncology departments of Abderrahmen Mami Hospital and Clinique Taoufik over a period of 17 years, between January 2000 and December 2016. Both hospitals encompass patients from the Tunisia north region. Patients with hematological malignancies were excluded. We collected patients diagnosed with BM and/or leptomeningeal carcinomatosis, either at initial diagnosis or during follow-up. BM diagnosis was based on either an imaging procedure, CT scan/MRI or on a histological specimen. We extracted the data about age, sex, primary tumor, clinical presentation and outcome from patient's records. We focused on populations of the two most common malignancies: breast and lung. This study was approved by the local ethical committee.

## Results

We identified 139 BM patients inside the 7055 cohort treated in both centers, which represented an incidence of 1.9%. Mean age at diagnosis was 54 years (28–86 years). Male patients were found to have BM more often (55.4%) than female patients with a sex ratio of 1.24. BM was secondary to a known primary tumor in 96.4% of cases. About two-thirds of BM (66.9%) were diagnosed during the course of the disease after a median time of 16 months (1–170 months) from initial primary cancer diagnosis. Most of the BMs were symptomatic at diagnosis (73.7%), expressed by focal signs, headaches and seizures accounting respectively for 29.7, 28.6 and 15.4%. Only two patients (1.8%) had associated upfront meningeal carcinomatosis. BM was the only site of metastasis in 36% of cases with a solitary lesion in 57.8% of cases. All of them had a CT scan and only 16 patients (11.5%) had an MRI. BM was unique in 80 patients (57.5%) and more than 3 in 25 cases (17.9%).

Patients were considered recursive partitioning analysis Class I in 36%, Class II in 36% and Class III in 29% of cases. Graded prognostic assessment (GPA) score was 0–1 in 26%, 1.5–2.5 in 52%, 3–9% and 3.5–4 in 13% of patients.

**Figure 1.**
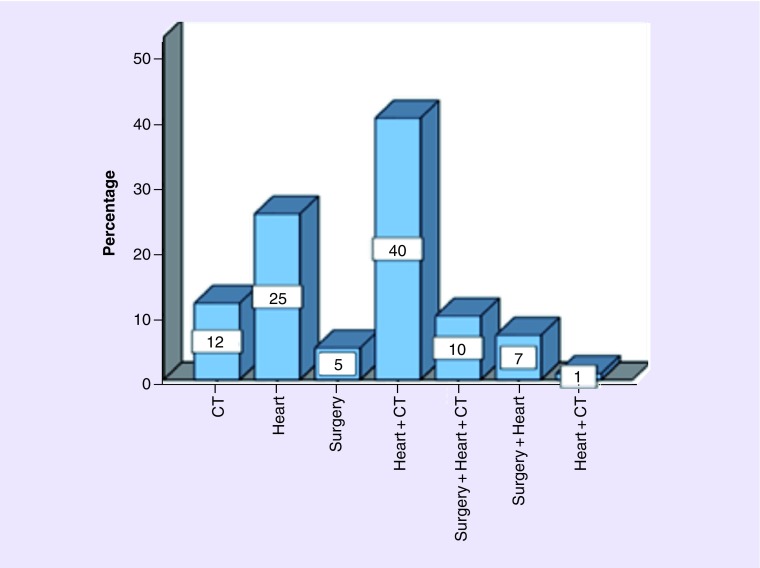
**Treatment approach for brain metastases in the overall population.** CT: Chemotherapy.

Therapy (local and/or systemic) was indicated in 73.4% of cases, and among them 88% received whole brain radiation therapy and 21.6% had metastasectomy (Figure 1).

**Figure 2.**
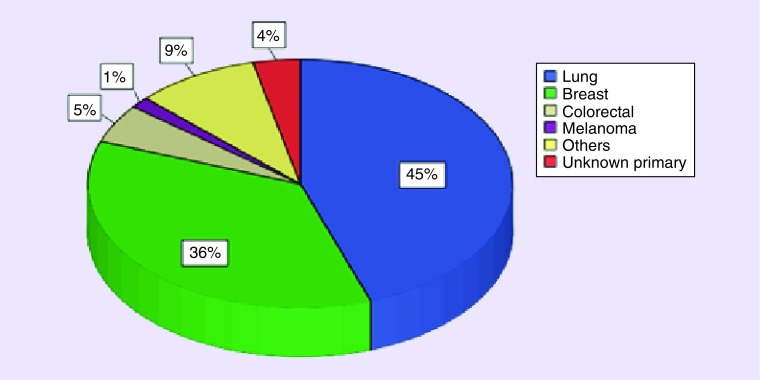
**Repartition of the primary tumors in brain metastases.**

In our series, lung (62 patients) and breast cancers (50 patients) represented 80% of the primary tumors. Whereas, colorectal and melanoma represented only 5 and 1.4% of primary malignancies (Figure 2). We detail characteristics of the most common locations: breast and lung.

Lung cancer was the most common primary with 62 cases out of 139 (45%). Patients were significantly older, compared with the other anatomic sites, with a mean age at diagnosis of 58 versus 50 years (p = 0.03). They were almost exclusively males in 93.5% of cases with a sex ratio at 14.5. BMs were detected at the moment of lung cancer diagnosis in 56.5% of cases, and among them 29.6% were asymptomatic. In patients with metachronous BM, the mean time from initial diagnosis of lung cancer to the occurrence of BM was 10 months. Extracranial involvement was found at diagnosis of BM in 22 patients (39.3%). Non-small-cell lung cancer (NSCLC) accounted for 75.8% of cases, adenocarcinoma presented 46.8% of cases, squamous cell carcinomas in 16.1% and undefined subtype in the remaining 12.9% of cases. NSCLCs were staged at presentation, according to TNM 2009 staging system, into stage IV in 71.1%, stage III in 23.7% and stages I–II in 5.2% of cases. Small cell lung cancer represented 27.3%, most of them had synchronous brain metastasis at diagnosis (60%). For small cell lung cancer and according to Veterans’ Affairs Lung Study Group staging system, limited stage was observed in 20% of cases. Only 6.7% of cases had previous prophylactic cerebral irradiation.

Breast cancer represented the second most common primary of BM with 50 cases out of 139 (36%). All patients were female and have a mean age at diagnosis of 49 years, significantly younger than the other locations (56 years, p = 0.02). Most of the patients presented with a metastatic (30%) or locally advanced (53%) stage at diagnosis. On histology, hormonal receptors were negative in 45% of cases and HER2 was overexpressed in 36% of cases. Almost all patients (94%) developed the BM during the course of their disease, with a mean time of 33.5 months (1–170 months). BMs were diagnosed in asymptomatic patients in 31.3% of cases. Patients had further extracranial localizations in 31 cases (67.4%). Modified breast-GPA was 1.5–2 in 33%, 2.5–3 in 50% and 3.5–4 in 17% of patients.

### Outcome

With a median follow-up of 33 months, median survival after BM diagnosis was 4 months. Lung cancer patients seemed to have a significant worse survival of 6 months, compared with breast cancer patients with 8 months (p = 0.022). Surgery of the BM showed a tendency to improved survival (11 vs 6 months; p = 0.548), but not statistically significant. Patients with synchronous BM had a nonsignificant tendency to a better survival than those with metachronous BM (synchronous, metachronous 11 vs 6 months; p = 0.284). We did not observe a difference in survival after BM between patients with only BM and patients with other extracranial metastases (6 vs 6 months; p = 0.696).

## Discussion

Our study concerning Tunisian patients focused on epidemiological patterns and outcome results of patients with BM and showed a predominance of lung and breast cancer primaries. To our knowledge, this is the largest reported population-based study concerning African patients. We believe that incidence reported in our series is underestimated for multiple reasons: difficult access to brain imaging procedures (especially MRI) during the first period of the study in our country, systematic brain imagery was not routinely performed, no autopsy was performed and the fact that this cohort is restricted to a ‘medical oncology’ department data and included also patients referred for indications of adjuvant–neoadjuvant or palliative chemotherapy. Historically reported population-based studies, such as Percy *et al*., that reviewed a period of 34 years showed an incidence rate of 11.1 per 100,000 per year [8]. Walker *et al*. conducted a surveillance, epidemiology and end results (SEERs) data-based survey concluding to an incidence rate of 8.3 per 100,000 patients admitted to short-term hospitals in the USA during 1973–1974 [9]. A more contemporary series from Barnholtz-Sloan *et al*. concerning the period from 1973 to 2001, found an incidence of 9.6% within a cohort of 16,210 patients [10]. Autopsy studies, usually reported higher incidence of BM, ranging from 9 to 26% [11,12]. However, even in autopsy series, incidence is also underestimated because CNS examination was not systematically carried out [13].

Consisting with our findings, two-third of brain tumors usually became symptomatic at presentation or during the course of the disease [14]. Most common symptoms are headaches, seizures and focal deficits accounting for 50, 20 and 40%, respectively [15,16]. Meningeal carcinomatosis was detected in only two of our 139 patients. As it is a well-known that the diagnosis of meningeal carcinomatosis is sometime challenging, it also represents a surrogate marker of bad prognosis with a median survival of 4 or 6 weeks [2]. Development of local therapies including whole brain radiation therapy, stereotactic radiosurgery and surgery raised the need for prognostic indexes to help select ‘curable’ patients. Therapeutic options in Tunisia are limited, external beam radiation therapy is the most available one, that is why therapy is not well developed. The Radiation Therapy Oncology Group developed simple tools to identify patients who are more likely to benefit from local treatment. The Recursive Partitioning Analysis, developed in 1997 [17], and the GPA, developed in 2008, are the most used ones. Disease-specific GPA index was afterward created such as the lung-GPA and breast-GPA were used to stratify patients in clinical trials [18,19]. Frequently, like our patients (56% of cases), the diagnosis of brain metastasis occurs concurrently to the diagnosis of lung cancer; however, there is a delay in metachronous metastasis diagnosis. In our population, BM occurred after an average of 10 months, unlike reported literature of an average of 19 months [13]. This again emphasizes the limited access to brain imaging leading to delay in diagnosis, in countries with limited resources, such as Tunisia.

Lung cancer and breast cancer were the most frequent primaries of BM in our results, in accordance with published data in which lung cancer accounts for 39–72% and breast cancer for around 17% [20]. Inside lung cancer population, the NSCLC subtype is predominant with 80–85% of cases, they are at 30–50% risk to develop BM versus up to 58% in small cell histologic [21]. Nowadays, it is recommended to perform in the initial work-up a cerebral imaging for small-cell lung and nonsquamous NSLC, due to the available local treatment methods (radiotherapy, radiosurgery and metastasectomy) that can improve survival and quality of life [22].

In the BM from breast cancer group, we observed younger age than the overall population, in accordance with literature data in which most cases are diagnosed in the fifth decade [23]. This is an expected result knowing the younger age at diagnosis of breast cancer in the Tunisian population (mean age at 51 years) [24]. Systematic brain imaging in CT scan work-up for breast cancer is a subject of debate. Considering the young age at which BMs are diagnosed, it may be considered even in asymptomatic patients in our context, however, survival benefit and cost–effectiveness should be studied to understand the relevance of such practice. Survival after BM diagnosis from breast cancer in our population was 8 months, which is in accordance with survival reported in western series [25].

BM incidence is likely underestimated with features similar to those reported in the literature and trends of younger age at diagnosis and better survival in breast cancer patients.
